# Assessing Historical Mining and Smelting Effects on Heavy Metal Pollution of River Systems over Span of Two Decades

**DOI:** 10.1007/s11270-017-3327-3

**Published:** 2017-03-13

**Authors:** Magdalena Strzebońska, Elżbieta Jarosz-Krzemińska, Ewa Adamiec

**Affiliations:** AGH University of Science and Technology, 30 Mickiewicza Ave., 30-059 Kraków, Poland

**Keywords:** Historical mining, River bottom sediment, Suspended particulate matter, Correlation coefficient

## Abstract

Research was conducted on the most polluted river system in Poland, impacted by active and historical mining. Bottom sediment, suspended particulate matter and river water were collected in 2014 from Przemsza river and its tributaries. Sampling points remained the same as those chosen in a 1995 study. This allowed the comparison of heavy metal accumulation in bottom sediment over a span of almost two decades. It was concluded that Przemsza river water and its tributaries are heavily contaminated with the following (in μg/dm^3^): Pb (0.99–145.7), Zn (48–5020), and Cd 0.12–12.72). Concentrations of metals in bottom sediment exceeded the background values by a factor of several hundred (100 times for Zn, 150 times for Pb, and 240 times for Cd). The arithmetic mean for metal concentration in fractions <63 μm sampled in 2014 has remained comparable to the level found in 1995 (in mg/kg): Zn 16,918 and 13,505, Pb 4177 and 4758, and Cd 92 and 134. It was determined that 20–50% more metals have accumulated in suspended matter, rather than in bottom sediment (in mg/kg): 20,498 Zn, Pb 5170, and 164 Cd. This exceeds the limits of the most polluted LAWA Class IV classification. Since the concentrations of Zn, Pb, and Cd increase drastically after the outlet of the Przemsza into the Vistula, it was concluded that river Przemsza is the cause of significant degradation of Vistula’s bottom sediment and suspended matter. A two-decade legacy of extremely high contamination of the Przemsza river sediments has persisted despite decreasing mining and smelting activity in the vicinity.

## Introduction

Multiple sources of mostly anthropogenic origin are causing pollution of aquatic environments with heavy metals. The most recognizable are current and historical mining and smelting industries, industrial effluents, leaks from dumping site leakage as well as common use of fertilizers and pesticides, or other surface runoffs. Heavy metals from atmospheric emissions also enter rivers. As indicated by Church and Scudlark (1998), they are responsible for the introduction of approximately 7% Cd, 4% Cu, 3% Zn, and 2% both Co and Ni into aquatic environments. Since heavy metals are persistent and not degradable under normal conditions, they pose a serious threat to aquatic environment even for hundreds of years after contamination. When introduced into rivers, they could be transported as compounds dissolved in water, be adsorbed onto suspended particulate matter, or be deposited in river bottom sediment. According to Martin and Meybeck 1979, almost 90% of contaminants are incorporated in the sediment-associated form rather than aqueous; this is mostly due to the sorptive nature of fine-grained suspended particles (Miller et al. 2007; Taylor and Hudson-Edwards 2008). Most riverbed sediments have fractions below 63 μm and are composed mainly of quartz as well as minor amounts of feldspar and carbonates. Finer fractions of sediments mostly compose of clay minerals, carbonates, or Mn and Fe hydroxides and oxides. The bottom sediment heavy metals are not permanently fixed but can be periodically remobilized, desorpted, or redistributed along the river course. Sediments act both as a sink and secondary source of metal contamination in surface water systems (Singh et al. 2005). The process of remobilization of heavy metals from both suspended particulate matter (SPM) and bottom sediment is potentially more dangerous than the accumulation itself. Mobility of contaminates is governed by changes of various physico-chemical parameters in which pH, redox potential, salinity, or the presence of complexation substances are of predominant concern. For instance, lowering pH by one unit can cause an increase of metal solubility by a factor of 10 (Förstner et al. 1990), which for example results in the dissolution of carbonates and hydroxides. Moreover, changing the redox condition from reducing to oxidizing also poses a great threat to the river environment, since it promotes transformation of stable metal sulfides into unstable metal sulfates. The use of river bottom sediment as environmental monitoring tool is often difficult as has been indicated by many authors (Regnier and Wollast 1993; Larsen et al. 1988; Eisma et al. 1989). Suspended particulate matter on the other hand was reported to be much better suited for monitoring purposes (Ji et al. 2016; Onderka and Pekárová 2008; Lartiges et al. 2001; Adamiec and Helios-Rybicka 2002). The role of suspended particulate matter in the uptake, release, and transport of heavy metals is obvious. Therefore, as stated by Turner and Millward 2002, SPM is a crucial link in heavy metal cycle between the water column, bed sediment, and consequently food chain. Assessing the quality of SPM provides the information about load of contaminants released from river bottom sediment but also from other more recent point sources of pollution. Contaminants are more likely immobilized in bottom sediment, and they are regarded as a potential source of contamination, in suspended matter; however, they are considered a real threat (Barbusiński et al. 2012). This component of the river system is also sensitive to the changes of physico-chemical parameters; for instance, lowering the pH (as a result of, e.g., active mining) promotes much easier transport of heavy metals from suspended matter into river water causing metals to become more available to living aquatic organisms and to further travel up to the end of the food chain.

River bottom sediment is commonly considered as a legacy of the past, especially where historical metal mining activity being the contamination source is concerned. Most studies regarding contamination of aquatic environment with heavy metals sourced from active or historical mining activities focus on examination of only bottom sediment (Jabłońska-Czapla et al. 2016; Dadová et al. 2016; Hogarh et al. 2016). However, in order to assess other than historical sources of river contamination and to differentiate between historical and present anthropogenic pollution sources, the investigation of suspended particulate matter should also be considered.

The research involved examining the bottom sediment, suspended particulate matter, and river water, collected from the Przemsza river and its tributaries in 2014. This is the most severely contaminated river in Poland. Its degradation can mostly be attributed to current and historical mining activities. The sampling points remained the same as these were chosen during sampling campaign in 1995 and carried out by authors (Helios-Rybicka et al. 1998). This allowed for a comparison of heavy metal accumulation in bottom sediment over a span of almost two decades. Moreover, an examination of suspended particulate matter was carried out in order to differentiate between recent sources of anthropogenic pollution and historical ones.

## Materials and Methods

### Study Area

**Fig. 1:**
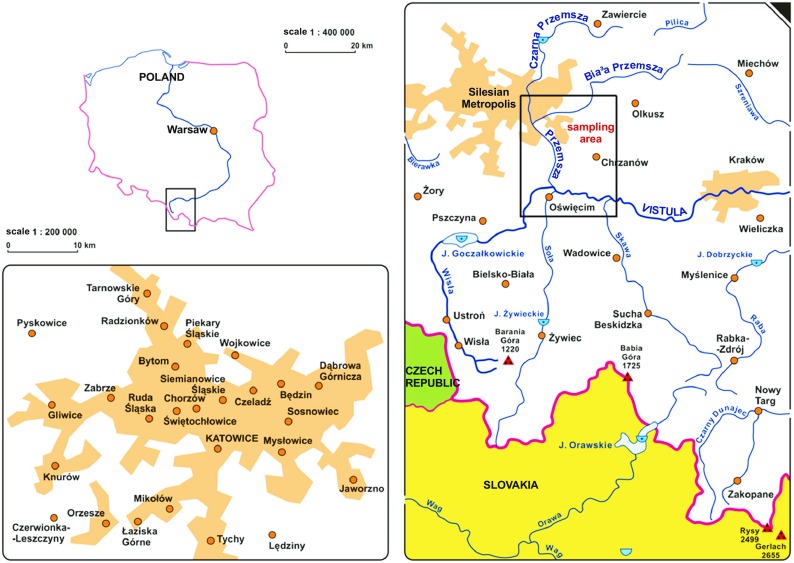
Research area (Olczyk 2016; source: www.tyflomapy.pl)

Przemsza river is one of the most polluted rivers in Poland and consequently in Europe. It comprises of Biała and Czarna Przemsza and its total length equals 87.6 km. It flows through highly industrialized and anthropogenically transformed area of the Upper Silesia, and it enters the Vistula river near Oświęcim (Auschwitz) (Fig. 1).

Multitude of factors of both natural and anthropogenic origin influences this river’s pollution with heavy metals. The most important ones are geological formations, lead-zinc and hard coal deposits, mining, industry, urbanization, and air pollution. Pb-Zn ores are deposited at the depth of 50 to 400 m and are accompanied by silver and cadmium deposits. This region is one of the biggest areas of zinc and lead deposits of so-called MVT (Mississippi Valley type), with parameters similar to deposits from the Mississippi River valley. The presence of these deposits led to the influx of population in the area of Upper Silesia and the rise of the largest and most densely populated agglomerations in Poland. These zinc-lead-silver ores are one of the oldest in Poland. They were mined in the area of Bytom from the Middle Ages, but now, they are mostly of historical significance.

Despite the fact that in this region, there is still approximately 80 million tons of zinc and lead ores, exploitation of only 12.5 million tons of these geological resources is profitable (data from 2010). The annual extraction rate of the last active mine—Pomorzany—is around 2.5 million tons of ore, and soon, the resources will be completely exhausted. Geochemical background of Upper Silesia region is naturally elevated since prehistorical times, and due to weathering, abnormally high concentrations of Zn, Pb, and Cd were recorded. However, according to the research of Klimek (1993), concentration of these metals spiked up in overbank sediments in the last 100–150 years. It was found that the amount of Zn, Cd, and Pb in the fraction <63 μm of overbank river sediments of the Przemsza river and its tributaries were one to two orders of magnitude higher when compared to the concentration of these metals determined in the layers of sediments before industrialization (anthropogenic pollution).

### Materials and Methods

Samples of river water and bottom sediments were collected from 13 locations along Przemsza and Vistula rivers and were stored in the polyethylene containers. Bottom sediments were sampled using probe from the depth of 20 cm. Physical parameters such as pH and conductivity were determined in situ in all samples*.* In the laboratory, samples of river water were filtered through <0.45 μm, acidified with nitric acid, and analyzed for heavy metal content using inductively coupled plasma-optical emission spectroscopy (ELAN 6100; Perkin Elmer). Samples of suspended matter, which were obtained as fraction <0.45 μm, were dried in 105 °C for 2 h and then digested with conc. HNO_3_ according to EPA 3051. Concentrations of Zn, Pb, and Cd were determined using ICP-MS, according to EN-ISO 17294-1 (2006). Bottom sediment samples were homogenized, mixed, and sieved through <63 μm; dried in 105 °C for 2 h; and furthermore, digested with conc. HNO_3_ according to EPA 3051. Concentrations of heavy metals were determined using atomic absorption spectroscopy (AAS) (F-AAS Thermo Scientific IC 3500).

## Results and Discussion

### River Water

Waters of Przemsza river and its tributaries are heavily contaminated with lead (0.99–145.7 μg/dm^3^), zinc (48–5020 μg/dm^3^), and cadmium (0.12–12.72 μg/dm^3^) (Table 1).

**Table 1:** Concentration of Zn, Pb, and Cd in water, suspended matter, and bottom sediment of the Przemsza river and its tributaries (2014 campaign)

River	Water (μg/dm^3^)			Suspended particulate matter (mg/kg)			Bottom sediment (mg/kg)		
	Zn	Pb	Cd	Zn	Pb	Cd	Zn	Pb	Cd
Biała	5020	8.57	12.72	39,097	21,848	340	21,822	8872	137
Biała Przemsza	48	6.90	0.27	2431	1206	37	765	670	13
Biała Przemsza	4076	11.92	10.26	34,666	18,170	269	28,158	7137	148
Sztoła	1570	145.70	3.37	26,211	9056	221	26,318	7033	167
Kozi Bród	249	23.65	0.68	8396	3064	71	2730	549	17
Biała Przemsza	1945	8.19	0.79	27,012	10,704	232	26,845	9451	154
Kanał Matylda	184	3.68	0.12	10,999	4112	79	7907	2976	64
Przemsza	698	1.10	0.32	19,881	4843	152	16,897	2133	93
Przemsza	821	1.34	0.27	20,587	5497	177	16,254	3051	74
Przemsza	667	0.99	0.19	20,409	4142	140	12,589	2620	67

The pH of the Przemsza river and its tributaries varies between 7.01 and 8.19 and does not exceed the threshold value corresponding to the first water quality class. Electrical conductivity ranges between 346 and 2395 μS/cm, but an average (median) values respond to the second purity class (Table 2).

**Table 2:** Statistical parameters of pH, conductivity, and concentration of Zn, Pb, and Cd in river water (2014 campaign)

Parameter	pH	Conductivity (μS/cm)	Zn (μg/dm^3^)	Pb (μg/dm^3^)	Cd (μg/dm^3^)
Biała Przemsza and Przemsza river					
Maximum	8.06	2395	4076	11.92	10.26
Minimum	7.50	346	48	0.99	0.19
Arithmetic mean	7.76	1404	1376	5.07	2.02
Median	7.73	1406	760	4.12	0.29
Geometric mean	7.75	1127	726	3.16	0.57
Przemsza river tributaries					
Maximum	8.19	1756	5020	145.70	12.72
Minimum	7.01	550	184	3.68	0.12
Arithmetic mean	7.55	931	1756	45.40	4.22
Median	7.49	708	910	16.11	2.03
Geometric mean	7.53	829	776	18.15	1.37
Vistula before Przemsza tributary					
	7.04	2690	153	9.13	0.54
Vistula after Przemsza tributary					
	7.09	3600	450	1.77	0.72

Zinc, lead, and cadmium content in river waters varies significantly and depends on the geological bedrock and the point sources of contamination. According to Kabata-Pendias and Pendias (1999), natural concentration of zinc in river water should be approximately 10 μg/dm^3^. Research revealed, however, that the average content of this element in Biała Przemsza and Przemsza waters was as high as 1406 μg/dm^3^. That is ten times more than the geochemical background established for the Upper Silesia rivers, which equals 86 μg/ dm^3^ (Pasieczna et al. 2010; Lis and Pasieczna 1995). Since lead compounds are poorly soluble at the pH around 7, the natural content of this element in river water should be as low as 0.2 μg/dm^3^ (Kabata-Pendias and Pendias 1999). In the Biała Przemsza and Przemsza river, however, an average concentration of Pb was found to be 4.12 μg/dm^3^, and it was lower than that previously reported in 2005 by Pasieczna et al. (2010) (median—34.3 μg/dm^3^). Natural background concentration of Cd in surface water should be as low as 0.02 μg/dm^3^ (Kabata-Pendias and Pendias 1999), but in both Biała Przemsza and Przemsza river, concentration of Cd was elevated and equaled to an average of 0.29 μg/dm^3^. Significant amount of Cd is introduced into those two rivers by its tributaries (median of 2.03 μg/dm^3^), especially by Biała river, which is the serious source of Cd contamination (12.7 μg/dm^3^).

These research findings are consistent with the chemical monitoring report provided in 2014 by the Regional Inspectorate of Environmental Protection in Katowice. It was reported that concentrations of priority substances and the so-called other impurities in the water exceeded the quality standards with respect to cadmium and lead (Biała river, Biała Przemsza from Ryczówka to Kozi Bród and from Kozi Bród to estuary); hexachlorocyclohexane and the sum of aldrin, dieldrin, endrin, and isodrin (Przemsza from Biała Przemsza to the estuary) as well as PAHs (Biała Przemsza, from Kozi Bród to the estuary and Przemsza, from Biała Przemsza to the estuary).

### Suspended Particulate Matter

Suspended particulate matter of the Przemsza river and its tributaries is severely contaminated with all of the investigated heavy metals (Tables 1 and 3). Concentrations of metals vary within sampling points (2431–39,097 mg/kg of Zn, 1206–21,898 mg/kg of Pb, and 37–340 mg/kg of Cd) reaching the highest levels in the suspended matter from the Biała tributary.

**Table 3:**
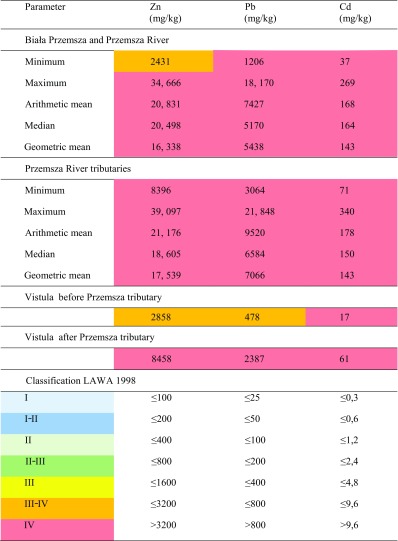
Statistical parameters of Zn, Pb, and Cd concentration in suspended matter of the Przemsza, its tributaries, and Vistula river (2014 campaign). Classification of suspended matter according to LAWA

Despite the fact that suspended matter plays a very important role in the transport of heavy metals in the river system, Polish regulations do not specify legal requirements for its quality. For the purpose of this study, comparison with threshold concentrations for individual metals, set by German guidelines—LAWA, was employed (Irmer 1997; LAWA 1998) (Table 3).

The results revealed that SPM collected from almost all sampling locations along the Przemsza rivers and its tributaries is exceeding the threshold values for the most polluted Class IV according to LAWA classification with respect to Zn, Pb, and Cd concentration. Only samples from the beginning coarse of the Biała Przemsza river contain an amount of Zn corresponding to the class III–IV, and the sample from the Vistula river before the outlet to Przemsza could be assigned to III purity class with respect to Pb contamination.

It was concluded that the Przemsza’s river suspended matter is excessively more contaminated with heavy metals than Oder river, the second biggest and cross-border river in Poland. Concentrations of heavy metals reported by Adamiec (2004) in 2000 in the Oder river’s suspended matter were found to be 1618 mg/kg Zn, 64.5 mg/kg Pb, and 9.24 mg/kg Cd.

### River Bottom Sediment

**Fig. 2:**
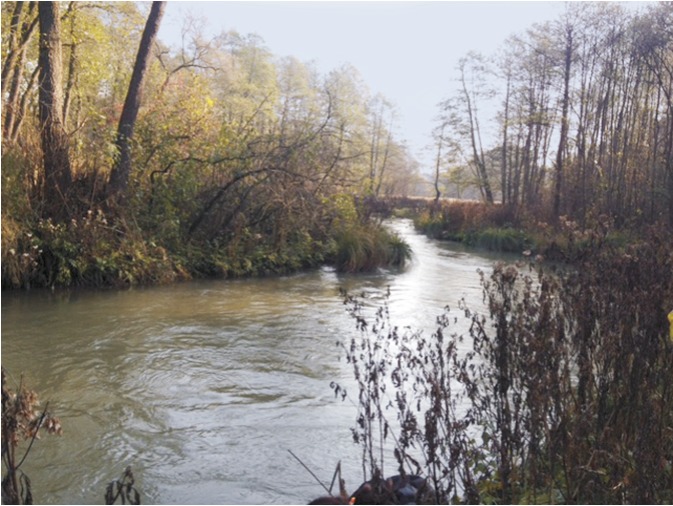
Biała Przemsza river after Biała river tributary

Bottom sediment of the Przemsza river and its tributaries is severely contaminated with heavy metals exceeding hundreds of times the background values established by Turekian & Wedepohl 1961 (Table 4). Maximum metal concentrations in the fraction <63 μm sampled in 2014 and in 1995 were as follows respectively (in mg/kg): Zn 28,158 and 29,580, Pb 9451 and 19,510, and Cd 154 and 269. The arithmetic mean for concentration of Zn, Pb and Cd in the fraction <63 μm of all investigated rivers has remained comparable in both collection periods and equals respectively to the following (in mg/kg): Zn 16,918 and 13,505, Pb 4177 and 4758, and Cd 92 and 134. Very high load of metals is introduced to the Przemsza river by its tributaries. In 2014, the concentrations of heavy metals in the bottom sediment of the most polluted rivers Biała Przemsza (Fig. 2) and Sztola were as high as follows (in mg/kg): Zn 28,158 and 26,318, Cd 154 and 167, and Pb 9451 and 7033.

**Table 4:**
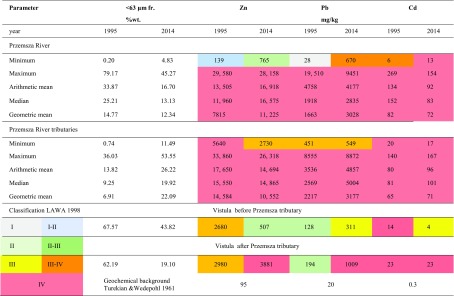
Statistical parameters of Zn, Pb, and Cd concentration in bottom sediment of the Przemsza, its tributaries, and Vistula river. Classification of bottom sediment according to LAWA

The highest contamination of bottom sediment with Pb was reported in Biała river tributary (8872 mg/kg). The geometric averages for concentration of Zn, Pb, and Cd are comparable for the Przemsza river and its tributaries, and they are found to be respectively as follows (in mg/kg): Zn 11,225 and 10,552, Pb 30,120 and 3177, and Cd 72 and 71. Concentrations of metals in all investigated bottom sediment samples are exceeding geochemical background concentrations established by Turekian and Wedepohl (1961) by a hundredfold or more: 100 times for Zn, 150 times for Pb, and for 240 times with respect to Cd.

Research revealed that bottom sediment of the Przemsza river and its tributaries has been classified as severely contaminated with heavy metals according to LAWA IV purity class. Only Biała Przemsza in its initial run, before the estuary of the Biała river, and river Kozi Bród are less contaminated with zinc and lead. Nonetheless, outlet of the Przemsza into Vistula river has caused the deterioration of its bottom sediment to IV quality class with respect to all metals.

### Correlation Calculated for All Components of the River System

**Fig. 3:**
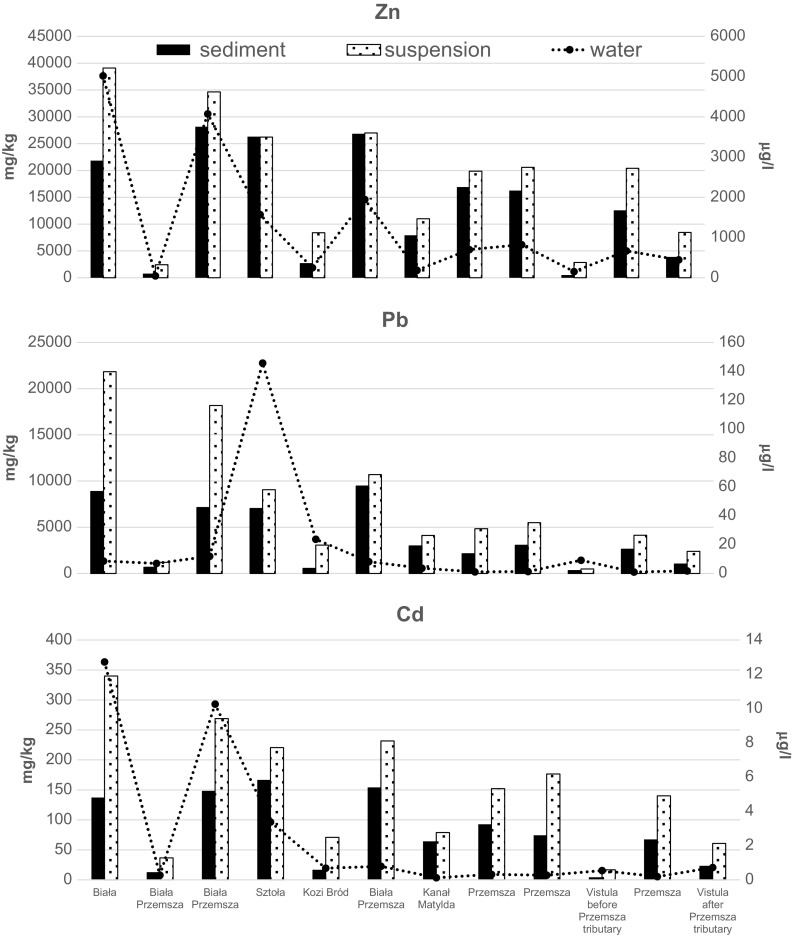
Comparison of Zn, Pb, and Cd concentrations in the bottom sediment, suspended matter, and river water along the Biała Przemsza and Przemsza rivers

Comparison of the concentrations of individual metals in the bottom sediment, suspended matter, and river water along the Biała Przemsza and Przemsza rivers (Fig. 3) revealed that there is a strong correlation between all components of the river system. The correlation coefficients calculated for each pair of variables showed strong, very strong, or almost full correlation for most couples, and the average correlation for only five pairs of variables (Table 5). This proves the existence of the same sources of heavy metal contamination for the examined rivers. Elements coexist with each other, and their content in the sediment, water, and the suspension is distributed proportionally along the entire course of Biała Przemsza and Przemsza rivers.

**Table 5:** Correlation coefficients for river water, suspended particulate matter, and bottom sediment of the Przemsza and Biała Przemsza rivers

	Sed. Zn	Sed. Pb	Sed. Cd	Susp. Zn	Susp. Pb	Susp. Cd	Water Zn	Water Pb	Water Cd
Sed. Zn	–	0.885	0.988	0.963	0.879	0.987	0.831	0.426	0.665
Sed. Pb	0.885	–	0.923	0.784	0.812	0.854	0.763	0.631	0.475
Sed. Cd	0.988	0.923	–	0.927	0.870	0.955	0.821	0.488	0.557
Susp. Zn	0.963	0.784	0.927	–	0.884	0.982	0.853	0.352	0.655
Susp. Pb	0.857	0.812	0.870	0.884	–	0.904	0.995	0.540	0.885
Susp. Cd	0.987	0.854	0.955	0.982	0.904	–	0.864	0.427	0.641
Water Zn	0.831	0.763	0.821	0.853	0.995	0.864	–	0.884	0.982
Water Pb	0.426	0.631	0.488	0.352	0.540	0.427	0.884	–	0.904
Water Cd	0.655	0.475	0.557	0.655	0.855	0.641	0.982	0.904	–

## Conclusions

The 2014 study revealed that Przemsza river is the cause of a significant increase in metal contamination in both river bottom sediment and suspended particulate matter of Poland’s main river, the Vistula. Determined concentrations of Zn, Pb and Cd increased drastically downstream from the outlet of the Przemsza to the Vistula river. All of the Przemsza’s river components, water, suspended particulate matter, and bottom sediment, are severely contaminated with heavy metals. Extremely high concentrations of metals in the Przemsza river bottom sediments persisted for over two decades on the comparable level or even higher even despite of decrease in activity of mining and metal casting industries in the vicinity. The arithmetic mean of metal concentration in size fraction <63 μm in 1995 and 2014 (in mg/kg) was as follows respectively: Zn 16,918 and 13,505, Pb 4177 and 4758, and Cd 92 and 134. Results confirm other authors’ reports (Graham Bird 2016; Audry et al. 2004) that elevated concentrations of heavy metals in river sediments continue to persist in river catchments despite mining having ceased for over 10 to a 100 years, sometimes even exceeding guideline concentrations hundreds of times. Przemsza’s bottom sediment is enriched with heavy metals exceeding geochemical background values established by Turekian and Wedepohl (1961): 100 times in terms of Zn, 150 times for Pb, and for 240 times with respect to Cd. However, it turns out that even more heavy metals are accumulated in suspended particulate matter, up to 20–50% more than in the river bottom sediment. This is in line with the findings of other authors’ claims that heavy metals are transported in the river system mostly bound with the suspended sediment. Such high concentrations of heavy metals in the suspended particulate matter are not only the resultant of their release from bottom sediments but must also be attributed to other constant point sources of pollution. Regardless of whether we take into account the arithmetic average, median, or geometric mean with regards to metal concentration in both suspended matter and bottom sediment, these parameters classify Przemsza river and its tributaries into the worst IV purity class according to the LAWA classification.
